# Thymoquinone: A Promising Therapeutic Agent for the Treatment of Colorectal Cancer

**DOI:** 10.3390/cimb46010010

**Published:** 2023-12-23

**Authors:** Natalia Kurowska, Marcel Madej, Barbara Strzalka-Mrozik

**Affiliations:** 1 Department of Molecular Biology, Faculty of Pharmaceutical Sciences in Sosnowiec, Medical University of Silesia, 40-055 Katowice, Poland; natalka.kurowska@gmail.com (N.K.); mmarcel281297@gmail.com (M.M.); 2 Silesia LabMed, Centre for Research and Implementation, Medical University of Silesia, 40-752 Katowice, Poland

**Keywords:** *Nigella sativa*, thymoquinone, colorectal cancer, adjuvant therapy, anticancer therapy, 5-FU resistance

## Abstract

Colorectal cancer (CRC) is one of the most commonly diagnosed cancers and is responsible for approximately one million deaths each year. The current standard of care is surgical resection of the lesion and chemotherapy with 5-fluorouracil (5-FU). However, of concern is the increasing incidence in an increasingly younger patient population and the ability of CRC cells to develop resistance to 5-FU. In this review, we discuss the effects of thymoquinone (TQ), one of the main bioactive components of *Nigella sativa* seeds, on CRC, with a particular focus on the use of TQ in combination therapy with other chemotherapeutic agents. TQ exhibits anti-CRC activity by inducing a proapoptotic effect and inhibiting proliferation, primarily through its effect on the regulation of signaling pathways crucial for tumor progression and oxidative stress. TQ can be used synergistically with chemotherapeutic agents to enhance their anticancer effects and to influence the expression of signaling pathways and other genes important in cancer development. These data appear to be most relevant for co-treatment with 5-FU. We believe that TQ is a suitable candidate for consideration in the chemoprevention and adjuvant therapy for CRC, but further studies, including clinical trials, are needed to confirm its safety and efficacy in the treatment of cancer.

## 1. Introduction

Colorectal cancer (CRC) is one of the most significant health problems worldwide [1,2]. It ranks third in the world in terms of cancer incidence and is the second leading cause of cancer deaths worldwide [3]. CRC is estimated to cause approximately one million deaths per year, and the 5-year and 10-year survival rates are 65% and 58%, respectively [1,2]. Approximately 41% of all colorectal cancers occur in the proximal colon, 22% in the distal colon and 28% in the rectum [4]. It has been suggested that in approximately 6–10% of cases, the incidence of CRC is associated with the presence of a known mutation in the genome [5]. In the majority of cases, colorectal cancer originates from precursor adenomatous or serrated polyps [2]. The development of colorectal cancer is the result of genetic, histological and morphological changes that accumulate over time. Depending on the origin of the mutations, CRC can be classified as familial, sporadic and hereditary [6].

Risk factors for CRC may be non-modifiable, such as genetic predisposition, the presence of inflammatory bowel disease or comorbidities (e.g., diabetes) and related hereditary conditions such as Lynch syndrome, familial adenomatous polyposis [1,5,6]. The incidence of CRC is also associated with the male sex and Asian and Black ethnicities [5]. However, environmental factors and a western lifestyle also contribute to the increased likelihood of developing this cancer, as the risk of CRC is higher in people with an abnormal body mass index, low physical activity, the consumption of processed meat and the abuse of stimulants (e.g., nicotine and high alcohol consumption) [1,5].

CRC is typically categorized into stages, ranging from stage 0 to stage IV. Stage 0 involves benign lesions, mainly non-metastatic polyps displaying hyperproliferative characteristics [7]. Stage I is characterized by malignant polyps, known as adenomas, which also affect the muscularis propria [7]. This stage often results from mutations in the *APC* gene. Stage II (early adenoma) and III occur when the tumor extends to the serous membrane or visceral peritoneum, respectively. Mutations in the *KRAS* oncogene are commonly observed in stage II, while stage III may additionally involve mutations in the *DCC* suppressor gene. In the final stage, stage IV, mutations in the *p53* gene occur, along with the development of distant metastases, primarily in the liver and lymphatic vessels. This stage is associated with a worse prognosis [7]. Treatment strategies for CRC vary depending on the stage, with adjuvant therapy often employed at each stage to improve outcomes [7].

**Figure 1:**
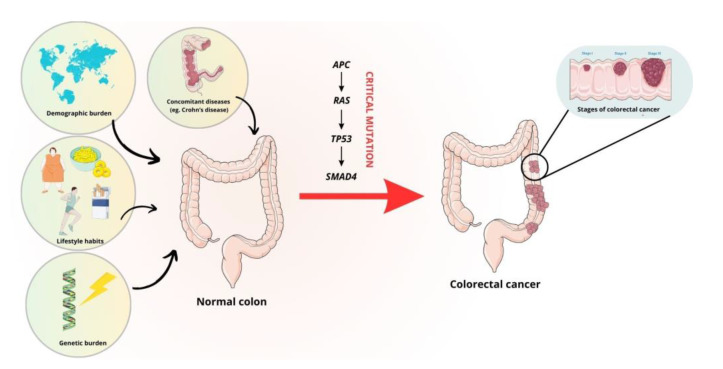
Risk factors and mutations involved in the development of CRC. The figure was partly generated using Servier Medical Art, provided by Servier and licensed under a Creative Commons Attribution 3.0 unported license.

The molecular background of carcinogenesis is extremely complex. It is known that CRC is a highly heterogeneous disease. The classical pathway of CRC development is associated with chromosomal instability, which is observed in 80% of CRCs [8]. It primarily involves mutations in the *APC* gene, which activates the WNT signaling pathway, which plays a major role in the development of CRC. Loss of function of the APC protein impairs the phosphorylation of β-catenin, which is not ubiquitinated and cannot be degraded by proteasomes. When β-catenin accumulates in excess, it is translocated to the nucleus where it works with the T-cell factor/lymphoid enhancer factor (LEF/TCF) protein complex to act as a transcription factor. This increases the expression of genes that stimulate cell growth [9,10]. Other molecular events include the activation of *KRAS* and *B-Raf proto-oncogene (BRAF)* mutations within the MAPK pathway [11,12]. Mutations within the mitogen-activated protein kinase (MAPK) pathway can also affect the gene encoding phosphoinositide 3-kinase (PIK3) [8,13]. In approximately 70% of cases, *tumor protein p53 (TP53)* loss-of-function mutations are associated with the classical pathway of carcinogenesis, resulting in the accumulation of the mutant protein in the nucleus [8,14]. Activation of the transforming growth factor β (TGF-β)-related pathway, mediated by a lack of expression of the effector protein SMAD family member 4 (SMAD4), is likely to be associated with the formation of distant metastases [8,15]. In addition, hypermethylation of CpG islands in the promoters of the genes involved in transcriptional regulation and microsatellite instability, mainly associated with mutations in the *BRAF* gene, the *TGF-β receptor type II* gene and the pro-apoptotic *bcl-2-like protein 4* (*BAX)* gene, may contribute to the development of CRC [8]. The modifiable and non-modifiable factors and critical mutations that influence the development of CRC are summarized in Figure 1.

Surgical resection of the tumor lesion or a larger part of the colon and chemotherapy are the mainstay of treatment for colorectal cancer. One of the most commonly used drugs is 5-fluorouracil (5-FU), which belongs to the group of pyrimidine antimetabolites [16]. The mechanism of antitumor action of 5-FU is mainly based on the inhibition of the enzyme thymidylate synthase, which leads to impaired DNA replication in cells. In addition, this chemotherapeutic agent can be incorporated into RNA, replacing approximately 50% of the uracil in the molecule, leading to impaired RNA synthesis. Both prodrugs and combinations of 5-FU with other chemotherapeutic agents are used in the treatment of CRC. A major limitation of this type of therapy is the ability of tumor cells to develop resistance to 5-FU [17].

In our opinion, the increase in the incidence of CRC in young people, i.e., under 50 years of age, and the emergence of resistance to standard treatment is particularly alarming [1,5,17]. For this reason, researchers are searching for new therapeutic regimens and alternatives. Several studies are currently focused on the effect of thymoquinone (TQ) on cancer cells. The aim of our work is to review current reports on the applicability and potential molecular targets of thymoquinone, a naturally derived compound, in the treatment and chemoprevention of colorectal cancer. In addition, we extended our review to include reports on the combination therapy of TQ with other chemotherapeutic agents currently in clinical use. For this purpose, we focused on the review of basic and preclinical studies in cellular and animal models.

## 2. *Nigella sativa*: The Main Source of Thymoquinone

Medicinal plants have been used since the dawn of time and are used in the preparation of herbal medicines because they are considered to be safer than modern allopathic drugs. One of these plants is *Nigella sativa*, commonly known as black seed or black cumin, which is native to southern Europe, North Africa and south-west Asia, and is grown in many countries around the world [18]. *Nigella sativa* is an annual plant that grows to a height of 45 cm and has long, lineal-lanceolate leaves that measure 2–2.5 cm. Flowering and fruiting occurs from January to April. *Nigella sativa* flowers are pale blue, 2–2.5 cm in diameter, and are solitary on long peduncles. Seeds of this plant are small dicotyledonous, trigonous, angular, regulose-tubercular, 2–3.5 × 1–2 mm, black on the outside and white inside [19]. *Nigella sativa* seeds contain several biologically active compounds, and their phytochemical composition varies depending on several factors, such as the growing region or maturity stage. Compounds isolated from *Nigella sativa* seeds belong to several chemical classes. The most notable is the family of terpenes and terpenoids, the most important of which is thymoquinone and its derivatives [20]. TQ accounts for about 25% of the volatile oil of *Nigella sativa* [21]. Other sources from which TQ is isolated are plants such as *Monarda didyma*, *Monarda media wild*, *Monarda menthifolia*, *Satureja hortensis*, *Satureja montana*, *Thymus pulegioides*, *Thymus serpyllum*, *Nepeta leucophylla*, *Tetraclinis articulata*, *Juniperus cedrus*, *Callitris quadrivalvis* and *Thymus vulgaris* [22,23,24]. However, *Nigella sativa* is the most commonly reported source of TQ in the literature [22]. The crude oil and thymoquinone extracted from its seeds and oil have many beneficial health properties that have been investigated in several research projects for their biological activity and potential therapeutic effects [18].

## 3. Characterization, Pharmacognostic Isolation and Purification of Thymoquinone

The molecular weight of TQ is 164.204 g/mol and its chemical formula is C_10_H_12_O_2_. TQ concentrations in seed oil have been reported between 18–25 µg/mL [24]. TQ is a member of the monoterpene class of compounds, which are formally derived from the condensation of two isoprene units. Secondary metabolism in plants leads to the formation of these compounds, which can be isolated using steam distillation or solvent extraction of plant parts [24]. Several methods have been described for the isolation and purification of TQ from plant material. These methods primarily involve supercritical fluid extraction, hydrodistillation, soxhlation and chromatographic techniques [23,25]. Using thin-layer chromatography on silica gel, the yellow crystalline molecule TQ was isolated [23]. Comparing two methods for the extraction of TQ from oil, the percentage obtained was 3% for extraction using hydrodistillation, as opposed to 48% for extraction using the Soxhlet method. A TQ-rich fraction was obtained using supercritical fluid extraction [23,26,27]. Ghanavi et al. [28] proposed a method for the isolation and purification of TQ from *Nigella sativa* seeds that has the potential for use in industrial applications, including a consideration in production for pharmaceutical purposes. They followed a two-step procedure for the TQ extraction: first, maceration with methyl tert-butyl ether, followed by liquid–liquid extraction with methanol, which effectively removed most of the impurities. Next, preparative high-performance liquid chromatography (HPLC) was performed to separate and purify the TQ. The results of the HPLC analysis showed that the purity of the collected TQ was 97%, while the gas chromatography–mass spectrometry results identified that the purity of the obtained TQ was 97%. Several studies concluded that organic solvent, supercritical CO_2_, or subcritical water extraction is a better method for separating TQ from herbal materials than water or steam distillation. Volatile compounds from *Monarda didyma* and *Monarda fistulosa* extracted with supercritical CO_2_ were much richer in TQ [29]. Other research found that the best solvent for TQ extraction from *Nigella sativa* was benzene, suggesting that the choice of the most efficient TQ extraction method may depend on the type of starting plant material [30]. There have also been published studies indicating that TQ is obtained from thymol by biotransformation using *Synechococcus* sp. However, the reported yield from this process was low [31]. Recently, the use of electrospun nanofibers as a sorbent for TQ extraction has been proposed by Nejabati et al. [32].

## 4. Properties and Pharmacological Features of Thymoquinone

TQ is present in tautomeric forms, such as the keto or enol form, or in mixtures of them [21,33]. Furthermore, due to its hydrophobic nature, the bioavailability and drug formulation of TQ are limited. In addition, the solubility of TQ in aqueous solutions varies from 549 to 669 µg/mL after 24 h to approximately 665 to 740 µg/mL after 72 h, and it depends on time [21,34]. The routes of administration of TQ include the oral, sub-acute, sub-chronic, intraperitoneal and intravenous routes [21,35]. After oral administration, liver enzymes may be elevated. This is due to metabolic activity, which reduces TQ to hydroquinone [21,36]. 

Toxicity data are also available for TQ, which are essential when considering its pharmaceutical potential. The lethal dose (LD_50_) varies depending on the route of administration, the carrier used and the model organism, but the LD_50_ of TQ after oral administration to mice was 870.9 mg/kg and 104.7 mg/kg after intraperitoneal injection [24,37]. A mean LD_50_ of 790 mg/kg and 57 mg/kg for oral and intraperitoneal administration, respectively, was reported for rats, with signs of toxicity, such as hypoactivity and respiratory depression [21,38]. TQ administered by intraperitoneal injection showed signs of toxicity associated with acute pancreatitis, and TQ administered orally showed signs of transient toxicity. Deaths were reported after doses of 500 mg/kg bw due to complications associated with intestinal obstruction. At the 300 and 500 mg/kg dose levels, signs of weight loss, diarrhea, mild abdominal distension and respiratory distress were observed in 34% of the rats within 48 h of administration. Subsequently, the rats regained weight and signs of toxicity began to disappear by the fifth day of the experiment [23,39]. In a study by Mansour et al. [40], rats and mice were given intraperitoneal injections at doses ranging from 5 mg/kg to 12.5 mg/kg and no toxicity was observed [40]. Other studies conducted by Kanter et al. [41] showed that the oral administration of 100 mg/kg or less of TQ also had no toxicological effect [41].

The pharmacokinetics of thymoquinone have been studied, using different dosing regimens of TQ administered by intraperitoneal, intravenous or intragastric routes, for their efficacy in disease models. Overall, it has been characterized that the usual doses of TQ tested are 5 mg/kg (intravenous) and 20 mg/kg (oral) [25]. This dose was used in a study by Alkharfy et al. [42] who investigated the plasma pharmacokinetic behavior of the intravenous and oral bioavailability of thymoquinone in a rabbit model. They found that the estimated clearance (CL) after intravenous administration was 7.19 ± 0.83 mL/kg/min and the volume of distribution at the steady state (Vss) was 700.90 ± 55.01 mL/kg. During the subsequent oral administration, the CL/F and Vss/F values were 12.30 ± 0.30 mL/min/kg and 5109.46 ± 196.08 mL/kg, respectively [42]. These parameters remained associated with an elimination half-life (t_1/2_) of 63.43 ± 10.69 and 274.61 ± 8.48 min for intravenous and oral administration, respectively [42]. The proposed t_1/2_ absorption was approximately 217 min. Compartmental analysis showed a t_1/2a_ of ~8.9 min and a t_1/2b_ of ~86.6 min. The predicted total bioavailability of TQ was maintained at ~58% for a lag time of ~23 min. They also found that binding to proteins was greater than 99% [42]. Alkharfy et al. [42] showed that TQ has high bioavailability, but oral administration is associated with rapid elimination and relatively slow absorption. Also, Ahmad et al. [39] used a dose of TQ 5 mg/kg intravenously and 20 mg/kg orally administered to rats. They found that after oral administration, the maximum plasma concentration (C_max_) of thymoquinone was 4.52 ± 0.092 μg/mL in male rats and 5.22 ± 0.154 μg/mL in female rats (*p* = 0.002). Similarly, after intravenous administration, the C_max_ was 8.36 ± 0.132 μg/mL in males and 9.51 ± 0.158 μg/mL in females (*p* = 0.550) [39]. The area under the plasma concentration–time curve after oral administration was 47.38 ± 0.821 μg/mL–h in females and 43.63 ± 0.953 μg/mL–h in males (*p* = 0.014) [39]. The results indicated that there were no sex differences in the pharmacokinetics of TQ.

The beneficial properties of thymoquinone are due to the presence of a lipophilic quinine moiety in its structure, which allows the molecule easy access to cellular and subcellular structures, as well as targeting intracellular transcription factors and kinases [43]. Despite the promising safety profile and high protein binding of TQ, its bioavailability is limited due to certain physicochemical properties. TQ is photosensitive and even brief exposure can lead to substantial degradation, whatever the solution acidity and solvent used [21]. Furthermore, TQ is unstable under alkaline conditions, with stability decreasing with increasing pH [34]. The solubility of TQ in aqueous media is <1.0 mg/mL at room temperature. In addition to its hydrophobic nature, TQ is characterized by slow absorption, rapid metabolism, rapid elimination and low physicochemical stability, which limits its pharmaceutical applications [23,24]. A study by Salmani et al. [34] shows a very low stability profile of TQ in all aqueous solutions, with rapid degradation that varied with the type of solvent. The study of degradation kinetics showed a significant effect of pH on the degradation process. Also, light sensitivity may limit the applicability of TQ [25]. To increase the bioavailability of TQ, researchers propose solutions based on the use of nanoformulations, i.e., liposomes, solid lipid nanoparticles, niosomes, nanostructured lipid carriers and nanoemulsions [24,44,45,46,47].

## 5. Preclinical and Clinical Studies on Thymoquinone and Its Main Source *Nigella sativa*

*Nigella sativa* has several promising pharmacological properties, mainly related to the presence of TQ, which has been confirmed in preclinical and clinical studies. Biologically active compounds from *Nigella sativa* have been shown to have antioxidant, antimicrobial, anti-inflammatory, antidiabetic, hepatoprotective, antiproliferative, proapoptotic, antiepileptic and immunomodulatory activities, among others [48,49,50,51,52,53,54]. Black seed could also be used for the prevention of some diseases, such as cardiovascular disease, diabetes, gastrointestinal diseases or wound healing [20,55,56,57,58,59]. In clinical studies, *Nigella sativa* and its constituents, including TQ, have demonstrated antimicrobial, antioxidant, anti-inflammatory, anticancer and anti-diabetic properties, as well as therapeutic effects on metabolic syndrome and gastrointestinal, neural, cardiovascular, respiratory, urinary and reproductive disorders [54]. Recent clinical trial reports suggest the potential use of *Nigella sativa* in complementary treatment and prevention of conditions such as coronary artery disease, diabetic peripheral neuropathy, *Helicobacter pylori* infection and polycystic ovary syndrome [60,61,62,63]. The safety of thymoquinone-rich black cumin oil was evaluated in a phase I clinical trial and provided satisfactory results [64]. Also, the antihyperglycemic activity of TQ was evaluated in a diabetic mouse model and patients [65]. Another pilot clinical trial proved that thymoquinone has anti-epileptic effects in children with refractory seizures [66]. 

Particular attention is currently being paid to research into the anticancer effects of TQ. Thymoquinone has anticancer properties that have been confirmed in preclinical studies of many types of cancer, including ovarian, colon, laryngeal, breast, leukemia, lung and osteosarcoma [67,68,69,70,71,72,73,74,75,76]. Currently, there is only one clinical trial evaluating the chemopreventive effect of thymoquinone on potentially malignant oral lesions in the clinicaltrials.gov database (accessed 14 November 2023). In addition, an Arabian phase I trial evaluated the clinical activity of thymoquinone in patients with advanced refractory malignant disease. TQ was found to be safe and well-tolerated in patients up to 10 mg/kg/day, but there was no significant anticancer activity found [77].

## 6. Brief on the Anticancer Properties of Thymoquinone

In vitro and in vivo studies have shown that TQ exerts tumorigenic effects in a variety of ways, including modulation of the epigenetic machinery and effects on proliferation, the cell cycle, apoptosis, angiogenesis, carcinogenesis and metastasis [43,78,79,80]. Thymoquinone also shows hepatoprotective and renal protective properties against drug cytotoxicity, affecting key enzymes involved in detoxification, i.e., aspartate transaminase, alanine transaminase, etc. [81]. It is also promising that TQ has low toxicity to normal cells, as confirmed by several studies, including studies on normal mouse kidney cells, normal human lung fibroblasts and normal human intestinal cells [82,83,84,85,86].

The ability of TQ to inhibit proliferation and influence apoptosis of cancer cells has been confirmed in studies on several cancer models, in addition to those mentioned above, this includes prostate and squamous cell carcinoma [87,88,89]. References show that TQ exerts its effect by influencing the expression of specific genes, including the upregulation of apoptotic mediators and the downregulation of transcription factors related to cytokine production [43]. TQ induces a pro-apoptotic effect by acting through numerous molecular mechanisms, such as the activation of c-Jun N-terminal kinases (JNK) and p38, as well as the phosphorylation of nuclear factor-κB (NF-κB) and the reduction of extracellular signal-regulated kinase 1/2 (ERK1/2) and phosphatidylinositol 4,5-bisphosphate 3-kinase (PI3K) activities [90,91]. TQ has also been shown to downregulate the PI3K/PTEN/Akt/mTOR and WNT/β-catenin pathways, which are critical for tumorigenesis [92,93]. TQ also interrupts metastasis by downregulating the epithelial to mesenchymal transition (EMT) transcription factors twist-related protein 1 (TWIST1) and E-cadherin [94]. For example, Zhang et al. [95] showed that TQ can suppress invasion and metastasis in bladder cancer cells by reversing EMT through the WNT/β-catenin pathway [95,96]. 

Oxidative stress is one of the factors in cancer development, as it leads to DNA damage [80]. The anticancer properties of TQ are also linked to its effects on oxidative stress, as TQ has been shown to act as an antioxidant at low concentrations. Higher concentrations, however, induce apoptosis of cancer cells through the induction of oxidative stress [90]. Thymoquinone acts by inducing cytoprotective enzymes, resulting in the protection of cells from cellular damage caused by oxidative stress. Thymoquinone upregulates the expression of genes encoding specific enzymes, such as catalase, superoxide dismutase, glutathione reductase, glutathione S-transferase and glutathione peroxidase, whose role is to protect against reactive oxygen species [43].

TQ has antitumor activity by reducing inflammation and acting as an immunomodulator. Inflammatory mediators and enzymes are known for their role in cancer development and progression [80]. TQ has the ability to downregulate NF-κB, interleukin-1β, tumor necrosis factor alpha, cyclooxygenase-2 (COX-2,) matrix metalloproteinase 13 (MMP-13), prostaglandin E2 (PGE2), the interferon regulatory factor, which are associated with inflammation and cancer development. What is more, TQ increases the activity of natural killer cells, which are essential in the body’s defense against cancer cells [43].

**Figure 2:**
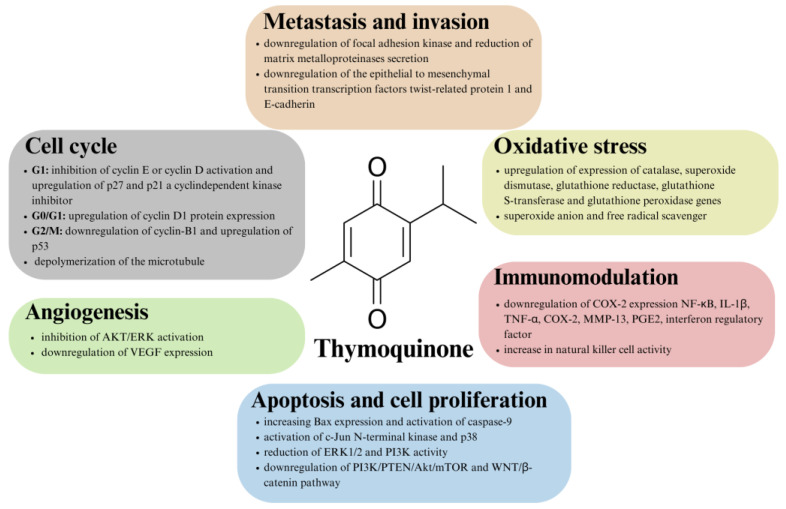
Overview of the mechanisms of action of thymoquinone in antitumor therapy.

Although the exact mechanism of TQ’s anticancer action is not known and fully characterized, it is generally possible to distinguish several points at which TQ acts. The general outline of the anticancer nature of thymoquinone is summarized in Figure 2.

## 7. Thymoquinone a Promising Candidate for the Treatment of Colorectal Cancer

### 7.1. Cytotoxic Effect of Thymoquinone on Colorectal Cancer Cells

The effect of thymoquinone on CRC has been studied in both cell lines and animal models. In general, the half-maximal inhibitory concentration (IC_50_) is the most commonly used indicator of a compound’s potency on cell viability and the evaluation of resistance. Available study reports were reviewed to determine whether TQ has the ability to inhibit CRC cells. The IC_50_ values of thymoquinone in selected colorectal cancer lines are summarized in Table 1.

Thymoquinone has shown the ability to inhibit CRC cells, demonstrating that it can be tentatively considered as a candidate for therapy or adjunctive treatment of CRC. In general, it has been observed that prolonged exposure to TQ results in a lower IC_50_. The effect exerted on cell lines varies, which may be related to the different genetic profile of each cell line and the effects of TQ on different molecular targets [103].

**Figure 3:**
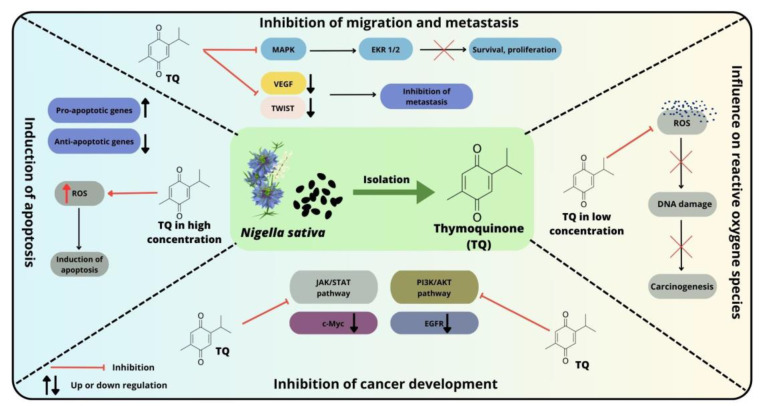
Selected mechanisms of action of thymoquinone against colorectal cancer cells and possible therapeutic targets.

Several pathways and molecular markers have been described that are significantly associated with the development and survival of colorectal cancer. In colon cancer cell lines, TQ exerts antitumorigenic effects through several mechanisms (Figure 3). Among the most commonly implicated pathways in CRC pathogenesis are the MAPK cascades downstream of the epidermal growth factor receptor (EGFR), TGF-β, JAK/STAT, TGF/SMAD, Notch, WNT/β-catenin, SHH/GLI, PI3K/AKT/mTOR and p53 pathways. The importance of these signaling pathways in the development and progression of CRC has been widely reported in the literature [95,104,105,106]. The results from recent studies indicate that these elements may be potential targets for TQ [107].

### 7.2. Influence of Thymoquinone on Apoptosis and Pathways Involved in Colorectal Cancer Cell Survival and Proliferation

Zhang et al. [108] investigated the antitumor potential of TQ on the colorectal cancer cell lines COLO205 and HCT-116. They showed that thymoquinone affected the reduction of tumor cell viability in a dose-dependent manner. They also investigated the molecular mechanism by which the antitumor effect was achieved. In summary, they showed that TQ exerts its antitumor effect by inhibiting NF-κB signaling, as TQ reduced the level of phosphorylated p65 in the nucleus, indicating the inhibition of NF-κB activation. In addition, the study showed that the expression of other genes related to oncogenesis, such as the *vascular endothelial growth factor* (*VEGF)*, *c-Myc* and *B-cell lymphoma 2 (Bcl-2)*, was reduced [108]. Similarly, Chen et al. [97] investigated the effect of TQ on irinotecan-resistant LoVo cells and obtained confirmation that it is an NF-κB inhibitor and demonstrated that TQ reduced the total and phosphorylated IκB kinase α (IKKα/β) and NF-κB and reduced metastasis in cancer cells. They also noted that TQ not only reduced the activity of ERK1/2 and PI3K, but also activated JNK and p38, which is associated with the suppression of metastasis. 

Kundu et al. [109] also investigated the effect of TQ on cells of the HCT-116 cell line and, like others, observed a decrease in cancer cell survival in the absence of TQ. They found that TQ affects the induction of apoptosis in cancer cells by blocking the signal transducer and activator of transcription 3 (STAT3) signaling via inhibition of Janus kinase 2 (JAK2) and Src-mediated phosphorylation of EGFR tyrosine kinase. They found that the induction of apoptosis was associated with the upregulation of *Bax* and inhibition of *Bcl-2* and *B-cell lymphoma-extra large* (Bcl-xl) expression, as well as activated caspase-9, -7 and -3, and induced cleavage of poly (ADP-ribose) polymerase (PARP). TQ also attenuated the expression of STAT3 target gene products, such as survivin, c-Myc and cyclin-D1, -D2, and enhanced the expression of cell cycle inhibitory proteins p27 and p21 [109]. 

Wirries et al. [110] studied the effect of TQ on colon cancer cells. Due to the low bioavailability of TQ, they decided to add saturated and unsaturated fatty acid residues to its molecule, thereby increasing its ability to penetrate membranes. They observed the most satisfactory results in terms of a decrease in tumor cell survival in response to treatment with thymoquinone-4-α-linolenoylhydrazone or thymoquinone-4-palmitoylhydrazone in the case of the p53-competent colon cancer line HCT-116. They found that the induced cytostatic effect, particularly in p53-competent HCT-116 cells, was mediated by the upregulation of p21^Cip1/Waf1^ and the downregulation of cyclin E and was associated with S/G2 arrest of the cell cycle. The fact that they did not observe such a strong effect in HCT-116p53^−/−^ cells confirms that the mechanism by which TQ derivatives inhibit cancer cell progression is dependent on the p53 status by activating the cell cycle inhibitor p21^Cip1/Waf1^ [110]. 

Hsu et al. [111] used a cell proliferation assay and an immunoblotting assay to study the effect of TQ on the migration of colon cancer cells from the LoVo cell line. The results confirmed that high doses of TQ significantly reduced the proliferation process of cancer cells. They found that TQ reduces the levels of p-PI3K, p-Akt, p-glycogen synthase kinase 3 (p-GSK3β) and β-catenin, thereby inhibiting downstream COX-2 expression, which in turn leads to a reduction in PGE2 levels and suppression of prostaglandin E2 receptor 2 (EP2) and prostaglandin E2 receptor 4 (EP4) activation. These studies have shown that the inhibition of COX-2 expression results in the reduced migration of colon cancer cells from the LoVo cell line. The effect of thymoquinone on cancer tumor growth has also been confirmed in an animal model [111]. Rooney et al. [112] studied the effects of two active compounds from *Nigella sativa* on the cells of various cancers, including the HT-29 colon cancer cell line. They showed that TQ dose-dependently inhibited the proliferation of HT-29 cells and induced their death, mainly by necrosis [112]. 

Two studies by the Gali-Muhtasib team involving HCT-116 colon cancer cells demonstrated that TQ induces apoptotic cell death in human colon cancer cells via a p53-dependent mechanism and that apoptosis in these cells is associated with the inhibition of the DNA damage sensor checkpoint kinase 1 (CHEK1) [113,114]. The results confirm that TQ can induce cancer cell death via different mechanisms. A study by Chen et al. [115] found that TQ induced cell death in the LoVo cell line via apoptosis, followed by mitochondrial outer membrane permeabilization and autophagic cell death. Most importantly, the treatment of colorectal cancer cells with TQ induced caspase-independent autophagic cell death via mitochondrial outer membrane permeabilization and the activation of JNK and p38 [115]. Animal models of carcinogenesis have also been used to investigate the potential anticancer effects of TQ on colon cancer cells. To investigate the effect of TQ, Asfour et al. [116] used 1,2-dimethylhydrazine (DMH)-induced colon carcinogenesis in a rat model. The results obtained by this research team appear to be very promising. They showed that a daily oral dose of TQ (10 mg/kg body weight) during the initiation phase had a chemopreventive effect against colon carcinogenesis. They also showed that TQ significantly prevented tumor formation (incidence), reduced tumor burden (multiplicity) and inhibited tumor growth [116].

### 7.3. Effect of Thymoquinone on Oxidative Stress-Related Damage in Colorectal Cancer

Oxidative stress has been strongly implicated in CRC by inducing mucosal inflammatory responses, genetic alterations, altered intestinal immune responses and changes in the composition of the intestinal microflora, which are considered to be an integral part of CRC carcinogenesis [117]. Research confirms the role of reactive oxygen species (ROS) in the initiation, progression and promotion of CRC [118,119,120]. Among other things, cellular damage may be associated with the activation of specific metabolic pathways and the induction of epigenetic changes [117]. To investigate the chemopreventive effects of *Nigella sativa* and its constituents, the team of Al-Johar et al. [121] used the azoxymethane (AOM)-induced colon cancer model in rats. They were able to show that *Nigella sativa* had an inhibitory effect on DNA damage in the test animals, but the exact mechanism of action could not be proven. The team attributed this to the probable antioxidant properties of *Nigella sativa* constituents, including thymoquinone [121]. 

The protective role of thymoquinone in colon carcinogenesis was also confirmed using DMH-induced colon carcinogenesis in the Wistar rat model. Jrah-Harzalach et al. [122] evaluated pre- and post-thymoquinone (TQ) treatment on DMH-induced oxidative stress during the initiation and promotion of colon carcinogenesis. Pretreatment with TQ completely reversed DMH-induced oxidative stress at the baseline, as well as histological changes and tumor progression. It also reversed oxidative damage during promotion and significantly reduced tumor incidence. In comparison, post-treatment with TQ corrected the oxidative status at the baseline and attenuated tumor development. These results support the potential use of thymoquinone in colorectal cancer chemoprevention, as TQ is effective in protecting and treating the DMH-initiated early phase of colorectal cancer. This effect is thought to be related to its antioxidant activity [122]. The Jrah-Harzalach research team also set out to determine the effects of thymoquinone on erythrocyte lipid peroxidation and the antioxidant status during DMH-induced colon carcinogenesis after initiation in male Wistar rats [122]. They showed that supplementation of the animals with thymoquinone restored DMH-induced oxidative stress markers to normal levels, confirming that TQ is a useful compound in preventing DMH-induced erythrocyte damage [123]. 

El-Najjar et al. [86] evaluated the effects of TQ on a panel of colorectal cancer cell lines, including Caco-2, HCT-116, LoVo, DLD-1 and HT-29, as well as normal human intestinal FHs74Int cells. They showed that TQ has the ability to kill cancer cells, while leaving normal cells unharmed. Furthermore, results from the DLD-1 line showed that the process of apoptosis in cancer cells is associated with the generation of ROS. They also found that TQ increased the phosphorylation states of MAPK, JNK and ERK [86].

Summarizing the results of the studies discussed above, two main modes of action can be distinguished in the activity of thymoquinone against CRC: a proapoptotic effect and the inhibition of proliferation, primarily related to the effect of TQ on the regulation of signaling pathways crucial in tumor progression, and oxidative stress. 

## 8. Simultaneous Stimulation with Thymoquinone and Known Chemotherapeutics: An Opportunity to Increase Treatment Efficacy

One of the major challenges in the treatment of colorectal cancer today is the development of resistance to the primary drug 5-FU. Resistance is estimated to occur in 90% of metastatic CRC cases [124]. The possible mechanisms of resistance to 5-FU are complex and varied and have been described extensively in the literature [124,125,126]. The most common are changes in the expression of genes encoding enzymes involved in the metabolism of 5-FU, particularly the upregulation of thymidylate synthase, the main enzyme [127]. In addition, cancer cells have the ability to regulate autophagy and apoptosis, for example by binding the active form of p53 and preventing its biological action, as well as modulating key signaling pathways, such as PI3K–AKT and MAPK–Ras–Erk [128,129]. Increased expression of TGF-β has also been associated with resistance to 5-FU [130]. CRC cells also cope with 5-FU by increasing 5-FU-activated ROS response mechanisms [124]. Other postulated mechanisms of resistance include multidrug resistance and membrane drug transporters, the influence of the tumor microenvironment and the occurrence of specific epigenetic changes in CRC cells [124,131,132,133,134]. Given the complexity and multiplicity of mechanisms of resistance to 5-fluorouracil in CRC, new therapeutic options are being sought to replace 5-FU or to overcome CRC cell resistance to 5-FU. Several studies confirming the feasibility of thymoquinone in combination therapy for CRC are discussed below.

Studies in an early-stage rat model of CRC showed that the co-administration of TQ and 5-FU resulted in a significant reduction in tumor size, greater than that seen with either treatment alone. In addition, the combination was found to decrease the expression of pro-tumor genes, such as *WNT*, *β-catenin*, *NF-κB*, *COX-2*, i*nducible *nitric oxide* synthase (iNOS)*, *VEGF*, and increase the expression of antitumor genes, namely *dickkopf-1 (DKK-1)*, *cyclin dependent kinase inhibitor 1A (CDNK-1A)*, *TGF-β1*, *TGF-βRII*, *Smad4* and *glutathione peroxidase* (*GPx)* [135]. Eftekhar and colleagues [136] reported similar findings. They showed that co-exposure of HT-29 cells to 5-FU and TQ resulted in greater cell inhibition than when the substances were used alone. They also showed that TQ has a synergistic effect on the pharmacokinetics of 5-FU [136]. TQ’s effect on CRC and cancer stem cells, considered the most resistant to 5-FU, has also been studied. It was shown that TQ significantly reduced the ability of cancer stem cells to regenerate and reduced the tumor size. This was attributed to DNA damage and the pro-apoptotic effect of TQ. Given that the results were also obtained in 5-FU-resistant cells, this suggests that TQ may be a promising adjuvant therapy [137]. In another study, the effect of a combination therapy of TQ and 5-FU on CRC using cancer stem cells was also investigated. The results suggest that the combination treatment is highly effective against cancer stem cell populations in CRC and simultaneously inhibits the WNT/β-catenin and PI3K/AKT signaling pathways, the most commonly implicated pathways in CRC recurrence and progression [138].

The effect of triple therapy with TQ, 5-FU and an additional agent on colorectal cancer cells was also studied. One trial also used vitamin D. Among the agents tested, TQ was shown to have greater antitumor activity than the other two, inducing apoptosis and showing higher expression of p21/p27/PTEN/BAX/Cyto-C/Casp-3 and increased levels of total glutathione, with inhibition of CCND1/CCND3/BCL-2 and PI3K/AKT/mTOR. Triple therapy, on the other hand, showed increased modulation of the PI3K/PTEN/Akt/mTOR pathway, higher expression of p21/p27/PTEN/BAX/Cyto-C/Casp-3 and better antioxidant effects than monotherapy [92]. Triple therapy with the addition of metformin was also studied. Three colorectal cancer lines were used in the study: HT-29, SW480 and SW620. Overall, the triple therapy proved to be the most effective, which was confirmed using molecular methods to induce cell cycle arrest and apoptosis in all cell lines. Nevertheless, each of the regimens, single, double and triple, achieved antitumor effects, but with less potency than the triple therapy [93]. Meanwhile, a comparative study investigated the efficacy of a combination of TQ and epigallocatechin-3-gallate (EGCG), one of the main active components of the green tea leaf, in the treatment of CRC. This work showed that the combination of these naturally occurring substances had a positive effect on CRC cells comparable to that of 5-FU. It was shown that the cell destruction and disruption of the cellular metabolic functions of human colorectal cancer cells was similar to that caused by exposure to 5-FU. At the same time, the morphological changes that occurred after exposure to TQ and EGCG were also comparable to cells exposed to 5-FU [139].

The use of TQ in the treatment of colorectal cancer may not only be an adjunct to 5-FU therapy, a substitute for it or chemoprevention. There is emerging evidence that TQ may also have a protective function, limiting the negative side effects of 5-FU on healthy cells. Zaki et al. [140] demonstrated that TQ in combination with onion extract has the ability to have a protective effect on liver cells. Animals supplemented with the extracts showed fewer pathological changes in liver cells and lower levels of liver enzymes than the control group [140]. Madani et al. [141] showed that thymoquinone protects against 5-FU-induced oral and intestinal mucositis in mice. These effects were attributed to the anti-inflammatory and antioxidant properties of TQ.

Moreover, 5-FU is not the only chemotherapy drug that can be used to treat CRC. However, the phenomenon of resistance affects many drugs, so new therapeutic agents are constantly being sought. The potential of thymoquinone has also been investigated in combination with compounds such as imatinib (IM), doxorubicin (DOX) and topotecan (TP). An overview of the studies is summarized in Table 2.

As shown above and confirmed by several studies in both animal and cellular models, thymoquinone has potential in the combination treatment of colorectal cancer. Thymoquinone can be used synergistically with chemotherapeutics, enhancing their anticancer effects and influencing the expression of signaling pathways and oncogenic pathways, but also protective genes, in cancer. There are also promising results indicating TQ’s potential against cancer stem cells. These data appear to be most relevant for co-treatment with 5-FU, as resistance to 5-FU is one of the major barriers to optimal treatment of colorectal cancer.

## 9. Future Directions

The antitumor potential of thymoquinone in CRC has been confirmed in animal and cellular models, and potentially possible molecular pathways of action appear to have been identified. However, there is still a need to expand the basic research to better understand which signaling pathways TQ acts on, which may prove useful in selecting targeted or adjuvant therapy for CRC patients. It is still necessary to accurately characterize the behavior of the drug, in the organism, including, but not limited to, pharmacokinetic/pharmacodynamic (PK/PD) modeling, as data on this topic are poor, and to develop the most efficient method for obtaining TQ. Nevertheless, further studies are required to confirm the clinical utility of TQ in CRC therapy. TQ has translational potential, as preclinical and clinical toxicity tests confirm its safety in humans. However, it can be a major challenge for the pharmaceutical industry to develop a convenient formulation of the drug with satisfactory pharmacokinetics and pharmacodynamics, due to problems with TQ’s bioavailability and rapid elimination. These challenges are being met by researchers who are developing new carriers for TQ that prolong its stability and enhance its safety. The final step, in the future, is to conduct clinical trials to confirm the safety and efficacy of TQ in the treatment of colorectal cancer.

## 10. Conclusions

Based on the available literature and published studies, it can be concluded that TQ has potential for use in the treatment of CRC due to its broad antitumor activity. Also in its favor is its high safety profile, low toxicity to normal cells and lack of information on significant side effects. The results of co-treating cancer cells with TQ and chemotherapeutic agents used in clinical practice also appear promising. Not only can TQ support therapy due to its synergistic effect with chemotherapeutics, but its ability to improve the sensitivity of CRC cells to chemotherapeutics has also been observed, which is curious in the case of 5-FU, since resistance to 5-FU is currently one of the biggest challenges in CRC treatment. Thus, the use of thymoquinone in chemoprevention and as an adjuvant therapy for CRC seems justified, especially because of its ability to offset the negative side effects of chemotherapy. However, more preclinical and clinical studies are needed to confirm the efficacy and safety of TQ in the treatment and chemoprevention of colorectal cancer. 

## Figures and Tables

**Table 1:** IC_50_ values of thymoquinone in selected colorectal cancer lines.

CRC Line	IC_50_ (µM)	Exposure Time (h)	Ref.
LoVo	6–8	24	[97]
C26	40	24	[98]
HT-29	112	24	[99]
DLD-1	61		
HCT-116	51.73	48	
HT-29	99.46		
HCT-15	82.59	24	[100]
HCT-116	68	24	[101]
HCT-116	20	24	[102]
	10.3	48	
	6.5	72	

CRC—colorectal cancer; IC_50_—half-maximal inhibitory concentration.

**Table 2:** Combination effect of thymoquinone with other chemotherapeutic agents in the treatment of colorectal cancer cell lines.

Chemotherapeutic Agent	Mechanism of Action	CRC Line	Effect of Combination Treatment	Ref.
IM	Protein tyrosine kinase inhibitor	HCT116	TQ-augmented cytotoxic activity of IM and synergized IM effect; TQ + IM decreases the expression of *ABCB1*, *ABCG2* and *hOCT1*; TQ + IM increases the uptake/efflux ratio of imatinib	[102]
DOX	DNA intercalation; Inhibitor of topoisomerase II; Generation of free radicals	HT-29	DOX + TQ was more effective than DOX alone against HT-29 cells desensitized by repeated DOX exposure	[142]
TP	Inhibitor of topoisomerase I	HT-29	Increased production of fragmented DNA; Inhibiting proliferation and lowering toxicity through p53- and Bax/Bcl2-independent mechanisms	[143]

CRC—colorectal cancer; IM—imatinib; TQ—thymoquinone; *ABCB1*—ATP-binding cassette subfamily B member 1; *ABCG2*—ATP-binding cassette super-family G member 2; *hOCT1*—human organic cation transporter 1; DOX—doxorubicin; TP—topotecan; Bax—Bcl-2 associated X-protein; Bcl2—B-cell lymphoma 2.

## Data Availability

Available on request and according to the regulations.

## References

[1] Li J., Ma X., Chakravarti D., Shalapour S., DePinho R.A. (2021). Genetic and biological hallmarks of colorectal cancer. Genes Dev..

[2] Sninsky J.A., Shore B.M., Lupu G.V., Crockett S.D. (2022). Risk factors for colorectal polyps and cancer. Gastrointest. Endosc. Clin. N. Am..

[3] Klimeck L., Heisser T., Hoffmeister M., Brenner H. (2023). Colorectal cancer. A health and economic problem. Best Pract. Res. Clin. Gastroenterol..

[4] Thanikachalam K., Khan G. (2019). Colorectal cancer and nutrition. Nutrients.

[5] Kanth P., Inadomi J.M. (2021). Screening and prevention of colorectal cancer. BMJ.

[6] Dariya B., Aliya S., Merchant N., Alam A., Nagaraju G.P. (2020). Colorectal cancer biology, diagnosis, and therapeutic approaches. Crit. Rev. Oncog..

[7] Hossain M.S., Karuniawati H., Jairoun A.A., Urbi Z., Ooi J., John A., Lim Y.C., Kibria K.M.K., Mohiuddin A.K.M., Ming L.C. (2022). Colorectal cancer: A review of carcinogenesis, global epidemiology, current challenges, risk factors, preventive and treatment strategies. Cancers.

[8] Bosman F., Yan P. (2014). Molecular pathology of colorectal cancer. Pol. J. Pathol..

[9] Disoma C., Zhou Y., Li S., Peng J., Xia Z. (2022). Wnt/β-catenin signaling in colorectal cancer: Is therapeutic targeting even possible?. Biochimie.

[10] Zhao H., Ming T., Tang S., Ren S., Yang H., Liu M., Tao Q., Xu H. (2022). Wnt signaling in colorectal cancer: Pathogenic role and therapeutic target. Mol. Cancer.

[11] Kundu S., Ali M.A., Handin N., Conway L.P., Rendo V., Artursson P., He L., Globisch D., Sjöblom T. (2021). Common and mutation specific phenotypes of KRAS and BRAF mutations in colorectal cancer cells revealed by integrative-omics analysis. J. Exp. Clin. Cancer Res. CR.

[12] Midthun L., Shaheen S., Deisch J., Senthil M., Tsai J., Hsueh C.T. (2019). Concomitant KRAS and BRAF mutations in colorectal cancer. J. Gastrointest. Oncol..

[13] Fang J.Y., Richardson B.C. (2005). The MAPK signalling pathways and colorectal cancer. Lancet. Oncol..

[14] Michel M., Kaps L., Maderer A., Galle P.R., Moehler M. (2021). The role of p53 dysfunction in colorectal cancer and its implication for therapy. Cancers.

[15] Papageorgis P., Cheng K., Ozturk S., Gong Y., Lambert A.W., Abdolmaleky H.M., Zhou J.R., Thiagalingam S. (2011). Smad4 inactivation promotes malignancy and drug resistance of colon cancer. Cancer Res..

[16] Mármol I., Sánchez-de-Diego C., Pradilla Dieste A., Cerrada E., Rodriguez Yoldi M. (2017). Colorectal carcinoma: A general overview and future perspectives in colorectal cancer. Int. J. Mol. Sci..

[17] Vodenkova S., Buchler T., Cervena K., Veskrnova V., Vodicka P., Vymetalkova V. (2020). 5-fluorouracil and other fluoropyrimidines in colorectal cancer: Past, present and future. Pharmacol. Ther..

[18] Ahmad A., Husain A., Mujeeb M., Khan S.A., Najmi A.K., Siddique N.A., Damanhouri Z.A., Anwar F. (2013). A review on therapeutic potential of Nigella sativa: A miracle herb. Asian Pac. J. Trop. Biomed..

[19] Rajsekhar S., Kuldeep B. (2011). Pharmacognosy and pharmacology of *Nigella sativa*—A review. Int. Res. J. Pharm..

[20] Hannan M.A., Rahman M.A., Sohag A.A.M., Uddin M.J., Dash R., Sikder M.H., Rahman M.S., Timalsina B., Munni Y.A., Sarker P.P. (2021). Black cumin (*Nigella sativa* L.): A comprehensive review on phytochemistry, health benefits, molecular pharmacology, and safety. Nutrients.

[21] Almajali B., Al-Jamal H.A.N., Taib W.R.W., Ismail I., Johan M.F., Doolaanea A.A., Ibrahim W.N. (2021). Thymoquinone, as a novel therapeutic candidate of cancers. Pharmaceuticals.

[22] Butt A.S., Nisar N., Ghani N., Altaf I., Mughal T.A. (2019). Isolation of thymoquinone from *Nigella sativa* L. and *Thymus vulgaris* L., and its anti-proliferative effect on HeLa cancer cell lines. Trop. J. Pharm. Res..

[23] Butnariu M., Quispe C., Herrera-Bravo J., Helon P., Kukula-Koch W., López V., Les F., Vergara C.V., Alarcón-Zapata P., Alarcón-Zapata B. (2022). The effects of thymoquinone on pancreatic cancer: Evidence from preclinical studies. Biomed. Pharmacother..

[24] Ahmad A., Mishra R.K., Vyawahare A., Kumar A., Rehman M.U., Qamar W., Khan A.Q., Khan R. (2019). Thymoquinone (2-Isoprpyl-5-methyl-1, 4-benzoquinone) as a chemopreventive/anticancer agent: Chemistry and biological effects. Saudi Pharm. J..

[25] Goyal S.N., Prajapati C.P., Gore P.R., Patil C.R., Mahajan U.B., Sharma C., Talla S.P., Ojha S.K. (2017). Therapeutic potential and pharmaceutical development of thymoquinone: A multitargeted molecule of natural origin. Front. Pharmacol..

[26] Burits M., Bucar F. (2000). Antioxidant activity of *Nigella sativa* essential oil. Phytother. Res..

[27] Solati Z., Baharin B.S., Bagheri H. (2012). Supercritical carbon dioxide (SC-CO_2_) extraction of *Nigella sativa* L. oil using full factorial design. Ind. Crops. Prod..

[28] Ghanavi Z., Velayati A.A., Frania P., Naji A.M., Kalatehjari S. (2020). Extraction and purification of anticancer thymoquinone from seeds of *Nigella sativa* by preparative high-performance liquid chromatography. JMPB.

[29] Sovova H., Sajfrtova M., Topiar M. (2015). Supercritical CO_2_ extraction of volatile thymoquinone from *Monarda didyma* and *M. fistulosa* herbs. J. Supercrit. Fluids.

[30] Iqbal M.S., Ahmad A., Pandey B. (2018). Solvent based optimization for extraction and stability of thymoquinone from *Nigella sativa* Linn. and its quantification using RP-HPLC. Physiol. Mol. Biol. Plants.

[31] Rasoul-Amini S., Fotooh-Abadi E., Ghasemi Y. (2011). Biotransformation of monoterpenes by immobilized microalgae. J. Appl. Physiol..

[32] Nejabati F., Ebrahimzadeh H. (2023). Electrospun nanofibers for extraction of thymoquinone from Nigella-Stevia prior to detection using electrochemical biosensor based on GCE/rGO/CuO. Microchem. J..

[33] Salem M.L. (2005). Immunomodulatory and therapeutic properties of the *Nigella sativa* L.. seed. Int. Immunopharmacol..

[34] Salmani J.M., Asghar S., Lv H., Zhou J. (2014). Aqueous solubility and degradation kinetics of the phytochemical anticancer thy-moquinone; probing the effects of solvents, pH and light. Molecules.

[35] Darakhshan S., Pour A.B., Colagar A.H., Sisakhtnezhad S. (2015). Thymoquinone and its therapeutic potentials. Pharmacol. Res..

[36] Nagi M.N., Almakki H.A. (2009). Thymoquinone supplementation induces quinone reductase and glutathione transferase in mice liver: Possible role in protection against chemical carcinogenesis and toxicity. Phytother. Res..

[37] Abukhader M.M. (2012). The effect of route of administration in thymoquinone toxicity in male and female rats. Indian J. Pharm. Sci..

[38] Rahmani A.H., Alzohairy M.A., Khan M.A., Aly S.M. (2014). Therapeutic implications of black seed and its constituent thymo-quinone in the prevention of cancer through inactivation and activation of molecular pathways. Evid. Based Complement Alternat. Med..

[39] Ahmad A., Alqahtani S., Jan B.L., Raish M., Rabba A.K., Alkharfy K.M. (2020). Gender effect on the pharmacokinetics of thymoquinone: Preclinical investigation and in silico modelling in male and female rats. Saudi Pharm. J..

[40] Mansour M.A., Ginawi O.T., El-Hadiyah T., El-Khatib A.S., Al-Shabanah O.A., Al-Sawaf H.A. (2001). Effects of volatile oil con-stituents of Nigella sativa on carbon tetrachloride-induced hepatotoxicity in mice: Evidence for antioxidant effects of thymo-quinone. Res. Commun. Mol. Pathol. Pharmacol..

[41] Kanter M. (2010). Thymoquinone attenuates lung injury induced by chronic toluene exposure in rats. Toxicol. Ind. Heal..

[42] Alkharfy K.M., Ahmad A., Khan R.M., Al-Shagha W.M. (2015). Pharmacokinetic plasma behaviors of intravenous and oral bioavailability of thymoquinone in a rabbit model. Eur. J. Drug Metab. Pharmacokinet..

[43] Mahmoud Y.K., Abdelrazek H.M.A. (2019). Cancer: Thymoquinone antioxidant/pro-oxidant effect as potential anticancer remedy. Biomed. Pharmacother..

[44] Elmowafy M., Samy A., Raslan M.A., Salama A., Said R.A., Abdelaziz A.E., El-Eraky W., El Awdan S., Viitala T. (2015). Enhancement of bioavailability and pharmacodynamic effects of thymoquinone via nanostructured lipid carrier (NLC) formulation. AAPS PharmSciTech.

[45] Khan A., Alsahli M.A., Aljasir M.A., Maswadeh H., Mobark M.A., Azam F., Allemailem K.S., Alrumaihi F., Alhumaydhi F.A., Alwashmi A.S.S. (2022). Safety, stability, and therapeutic efficacy of long-circulating TQ-incorporated liposomes: Implication in the treatment of lung cancer. Pharmaceutics.

[46] Hatami Nemati S., Bigdeli M.R., Mortazavi Moghadam F., Sharifi K. (2023). Neuroprotective effects of niosomes loaded with thymoquinone in the cerebral ischemia model of male Wistar rats. Nanomedicine.

[47] Alam M., Zameer S., Najmi A.K., Ahmad F.J., Imam S.S., Akhtar M. (2020). Thymoquinone loaded solid lipid nanoparticles demonstrated antidepressant-like activity in rats via indoleamine 2, 3-dioxygenase pathway. Drug Res..

[48] Manjunatha V., Nixon J.E., Mathis G.F., Lumpkins B.S., Güzel-Seydim Z.B., Seydim A.C., Greene A.K., Jiang X. (2023). Nigella sativa as an antibiotic alternative to promote growth and enhance health of broilers challenged with Eimeria maxima and Clostridium perfringens. Poult. Sci..

[49] Barashkova A.S., Smirnov A.N., Zorina E.S., Rogozhin E.A. (2023). Diversity of cationic antimicrobial peptides in black cumin (*Nigella sativa* L.) seeds. Int. J. Mol. Sci..

[50] Alam T., Naseem S., Shahabuddin F., Abidi S., Parwez I., Khan F. (2023). Oral administration of *Nigella sativa* oil attenuates arsenic-induced redox imbalance, DNA damage, metabolic distress, and histopathological alterations in rat intestine. J. Trace. Elem. Med. Biol..

[51] Ciesielska-Figlon K., Wojciechowicz K., Daca A., Kokotkiewicz A., Łuczkiewicz M., Witkowski J.M., Lisowska K.A. (2023). The impact of *Nigella sativa* essential oil on T cells in women with Hashimoto’s thyroiditis. Antioxidants.

[52] Ali Bakr E.H., Saad Alyamani R.A. (2023). Immunomodulatory protective effects of *Nigella sativa* and *Lactuca sativa* oils on liver intoxication in experimental animals. Pak. J. Biol. Sci..

[53] Younus H., Sawhney (2018). Molecular and Therapeutic: Actions of Thymoquinone.

[54] Gholamnezhad Z., Havakhah S., Boskabady M.H. (2016). Preclinical and clinical effects of *Nigella sativa* and its constituent, thymoquinone: A review. J. Ethnopharmacol..

[55] Dalli M., Bekkouch O., Azizi S.E., Azghar A., Gseyra N., Kim B. (2022). *Nigella sativa* L. phytochemistry and pharmacological activities: A review (2019–2021). Biomolecules.

[56] Jarmakiewicz-Czaja S., Zielińska M., Helma K., Sokal A., Filip R. (2023). Effect of *Nigella sativa* on selected gastrointestinal diseases. Curr. Issues Mol. Biol..

[57] Azami S., Forouzanfar F. Potential role of *Nigella sativa* and its constituent (thymoquinone) in ischemic stroke. Curr. Mol. Med..

[58] Alabdullah M., Kara Beit Z.Z., Shehada A. (2023). Comparative clinical study of the effect of *Nigella sativa* oil on soft tissue healing and inflammation reduction compared to Eugenol in the context of dry socket. Cureus.

[59] Palanisamy C.P., Alugoju P., Jayaraman S., Poompradub S. (2023). *Nigella sativa* L. seed extracts promote wound healing progress by activating VEGF and PDGF signaling pathways: An in vitro and in silico study. F1000Research.

[60] Tavakoli-Rouzbehani O.M., Abbasnezhad M., Kheirouri S., Alizadeh M. (2022). Efficacy of *Nigella sativa* oil on endothelial function and atherogenic indices in patients with coronary artery diseases: A randomized, double-blind, placebo-control clinical trial. Phytother. Res..

[61] Khodaie S.A., Nikkhah H., Namiranian N., Abotorabi M., Askari M., Khalilzadeh S.H., Khatibi Aghda A., Kamalinejad M. (2023). Topical *Nigella sativa* L. product: A new candidate for the management of diabetic peripheral neuropathy. Inflammopharmacology.

[62] Yousefnejad H., Mohammadi F., Alizadeh-Naini M., Hejazi N. (2023). *Nigella sativa* powder for helicobacter pylori infected patients: A randomized, double-blinded, placebo-controlled clinical trial. BMC Complement. Med. Ther..

[63] Balasubramanian R., Maideen N.M.P., Muthusamy S., Gobinath M. (2023). A review of clinical and preclinical studies on the therapeutic potential of black seeds (*Nigella sativa*) in the management of polycystic ovarian syndrome (PCOS). J. Pharmacopunct..

[64] Thomas J.V., Mohan M.E., Prabhakaran P., Das S.S., Maliakel B., Krishnakumar I.M. (2022). A phase I clinical trial to evaluate the safety of thymoquinone-rich black cumin oil (BlaQmax^®^) on healthy subjects: Randomized, double-blinded, placebo-controlled prospective study. Toxicol. Rep..

[65] Ali S.M., Chen P., Sheikh S., Ahmad A., Ahmad M., Paithankar M., Desai B., Patel P., Khan M., Chaturvedi A. (2021). Thymoquinone with metformin decreases fasting, post prandial glucose, and HbA1c in type 2 diabetic patients. Drug Res..

[66] Akhondian J., Kianifar H., Raoofziaee M., Moayedpour A., Toosi M.B., Khajedaluee M. (2011). The effect of thymoquinone on intractable pediatric seizures (pilot study). Epilepsy Res..

[67] Rajput S., Kumar B.P., Dey K.K., Pal I., Parekh A., Mandal M. (2013). Molecular targeting of Akt by thymoquinone promotes G1 arrest through translation inhibition of cyclin D1 and induces apoptosis in breast cancer cells. Life Sci..

[68] Şahin C., Maytalman E., Nemutlu Samur D., Doğan B. (2023). The effect of thymoquinone and propranolol combination on epidermoid laryngeal carcinoma cell. Eur. Arch. Otorhinolaryngol..

[69] Almajali B., Al-Jamal H.A.N., Wan Taib W.R., Ismail I., Johan M.F., Doolaanea A.A., Ibrahim W.N., Tajudin S.A. (2021). Thymoquinone suppresses cell proliferation and enhances apoptosis of HL60 leukemia cells through re-expression of JAK/STAT negative regulators. Asian Pac. J. Cancer Prev..

[70] Al-Rawashde F.A., Al-Sanabra O.M., Alqaraleh M., Jaradat A.Q., Al-Wajeeh A.S., Johan M.F., Wan Taib W.R., Ismail I., Al-Jamal H.A.N. (2023). Thymoquinone enhances apoptosis of K562 chronic myeloid leukemia cells through hypomethylation of SHP-1 and inhibition of JAK/STAT signaling pathway. Pharmaceuticals.

[71] Zhang Y., Liu X., Dang W., Liu L. (2023). Thymoquinone inhibits lung cancer stem cell properties via triggering YAP degradation. Carcinogenesis.

[72] Nithya G., Santhanasabapathy R., Vanitha M.K., Anandakumar P., Sakthisekaran D. (2023). Antioxidant, antiproliferative, and apoptotic activity of thymoquinone against benzo(a)pyrene-induced experimental lung cancer. J. Biochem. Mol. Toxicol..

[73] Sanapour N., Malakoti F., Shanebandi D., Targhazeh N., Yousefi B., Soleimanpour J., Majidinia M. (2022). Thymoquinone augments methotrexate-induced apoptosis on osteosarcoma cells. Drug Res..

[74] Khyavi P.A., Valizadeh A., Shanehbandi D., Yousefi B., Soleimanpour J. (2022). Thymoquinone potentiates methotrexate mediated-apoptosis in Saos-2 osteosarcoma cell line. Drug Res..

[75] Liu X., Dong J., Cai W., Pan Y., Li R., Li B. (2017). The effect of thymoquinone on apoptosis of SK-OV-3 ovarian cancer cell by regulation of Bcl-2 and Bax. Int. J. Gynecol. Cancer.

[76] Khurshid Y., Syed B., Simjee S.U., Beg O., Ahmed A. (2020). Antiproliferative and apoptotic effects of proteins from black seeds (*Nigella sativa*) on human breast MCF-7 cancer cell line. BMC Complement. Med. Ther..

[77] Al-Amri A.M., Bamosa A.O. (2009). Phase I safety and clinical activity of thymoquinone in patients with advanced refractory malignant disease. Shiraz. E-Med. J..

[78] Khan M.A., Tania M., Fu J. (2019). Epigenetic role of thymoquinone: Impact on cellular mechanism and cancer therapeutics. Drug Discov. Today.

[79] Ansary J., Giampieri F., Forbes-Hernandez T.Y., Regolo L., Quinzi D., Gracia Villar S., Garcia Villena E., Tutusaus Pifarre K., Alvarez-Suarez J.M., Battino M. (2021). Nutritional value and preventive role of *Nigella sativa* L. and its main component thymoquinone in cancer: An evidenced-based review of preclinical and clinical studies. Molecules.

[80] Alhmied F., Alammar A., Alsultan B., Alshehri M., Pottoo F.H. (2021). Molecular mechanisms of thymoquinone as anticancer agent. Comb. Chem. High Throughput Screen..

[81] Khan M.A., Younus H. (2018). Thymoquinone shows the diverse therapeutic actions by modulating multiple cell signaling pathways: Single drug for multiple targets. Curr. Pharm. Biotechnol..

[82] Badary O., Al-Shabanah O.A., Nagi M.N., Al-Bekairi A.M., Elmazar M.M. (1998). Acute and subchronic toxicity of thymoquinone in mice. Drug Dev. Res..

[83] Abukhader M.M. (2013). Thymoquinone in the clinical treatment of cancer: Fact or fiction?. Pharmacogn. Rev..

[84] Park E.J., Chauhan A.K., Min K.J., Park D.C., Kwon T.K. (2016). Thymoquinone induces apoptosis through downregulation of c-FLIP and Bcl-2 in renal carcinoma Caki cells. Oncol. Rep..

[85] Gurung R.L., Lim S.N., Khaw A.K., Soon J.F., Shenoy K., Mohamed Ali S., Jayapal M., Sethu S., Baskar R., Hande M.P. (2010). Thymoquinone induces telomere shortening, DNA damage and apoptosis in human glioblastoma cells. PLoS ONE.

[86] El-Najjar N., Chatila M., Moukadem H., Vuorela H., Ocker M., Gandesiri M., Schneider-Stock R., Gali-Muhtasib H. (2010). Reactive oxygen species mediate thymoquinone-induced apoptosis and activate ERK and JNK signaling. Apoptosis.

[87] Kou B., Liu W., Zhao W., Duan P., Yang Y., Yi Q., Guo F., Li J., Zhou J., Kou Q. (2017). Thymoquinone inhibits epithelial-mesenchymal transition in prostate cancer cells by negatively regulating the TGF-β/Smad2/3 signaling pathway. Oncol. Rep..

[88] Karimian A., Majidinia M., Moliani A., Alemi F., Asemi Z., Yousefi B., Fazlollahpour Naghibi A. (2022). The modulatory effects of two bioflavonoids, quercetin and thymoquinone on the expression levels of DNA damage and repair genes in human breast, lung and prostate cancer cell lines. Pathol. Res. Pract..

[89] Chu S.C., Hsieh Y.S., Yu C.C., Lai Y.Y., Chen P.N. (2014). Thymoquinone induces cell death in human squamous carcinoma cells via caspase activation-dependent apoptosis and LC3-II activation-dependent autophagy. PLoS ONE.

[90] Imran M., Rauf A., Khan I.A., Shahbaz M., Qaisrani T.B., Fatmawati S., Abu-Izneid T., Imran A., Rahman K.U., Gondal T.A. (2018). Thymoquinone: A novel strategy to combat cancer: A review. Biomed. Pharmacother..

[91] El-Far A.H., Tantawy M.A., Al Jaouni S.K., Mousa S.A. (2020). Thymoquinone-chemotherapeutic combinations: New regimen to combat cancer and cancer stem cells. Naunyn Schmiedebergs Arch. Pharmacol..

[92] Idris S., Refaat B., Almaimani R.A., Ahmed H.G., Ahmad J., Alhadrami M., El-Readi M.Z., Elzubier M.E., Alaufi H.A.A., Al-Amin B. (2022). Enhanced in vitro tumoricidal effects of 5-Fluorouracil, thymoquinone, and active vitamin D3 triple therapy against colon cancer cells by attenuating the PI3K/AKT/mTOR pathway. Life Sci..

[93] Farrash W.F., Aslam A., Almaimani R., Minshawi F., Almasmoum H., Alsaegh A., Iqbal M.S., Tabassum A., Elzubier M.E., El-Readi M.Z. (2023). Metformin and thymoquinone co-treatment enhance 5-fluorouracil cytotoxicity by suppressing the PI3K/mTOR/HIF1α pathway and increasing oxidative stress in colon cancer cells. Biofactors.

[94] Fatfat Z., Fatfat M., Gali-Muhtasib H. (2021). Therapeutic potential of thymoquinone in combination therapy against cancer and cancer stem cells. World J. Clin. Oncol..

[95] Zhang M., Du H., Wang L., Yue Y., Zhang P., Huang Z., Lv W., Ma J., Shao Q., Ma M. (2020). Thymoquinone suppresses invasion and metastasis in bladder cancer cells by reversing EMT through the Wnt/β-catenin signaling pathway. Chem. Biol. Interact..

[96] Koveitypour Z., Panahi F., Vakilian M., Peymani M., Forootan F.S., Esfahani M.H.N., Ghaedi K. (2019). Signaling pathways involved in colorectal cancer progression. Cell Biosci..

[97] Chen M.C., Lee N.H., Hsu H.H., Ho T.J., Tu C.C., Chen R.J., Lin Y.M., Viswanadha V.P., Kuo W.W., Huang C.Y. (2017). Inhibition of NF-κB and metastasis in irinotecan (CPT-11)-resistant LoVo colon cancer cells by thymoquinone via JNK and p38. Environ. Toxicol..

[98] Gali-Muhtasib H., Ocker M., Kuester D., Krueger S., El-Hajj Z., Diestel A., Evert M., El-Najjar N., Peters B., Jurjus A. (2008). Thymoquinone reduces mouse colon tumor cell invasion and inhibits tumor growth in murine colon cancer models. J. Cell Mol. Med..

[99] Al Bitar S., Ballout F., Monzer A., Kanso M., Saheb N., Mukherji D., Faraj W., Tawil A., Doughan S., Hussein M. (2022). Thymoquinone radiosensitizes human colorectal cancer cells in 2D and 3D culture models. Cancers.

[100] Osorio-Pérez S.M., Estrada-Meza C., Ruiz-Manriquez L.M., Arvizu-Espinosa M.G., Srivastava A., Sharma A., Paul S. (2023). Thymoquinone potentially modulates the expression of key onco- and tumor suppressor miRNAs in prostate and colon cancer cell lines: Insights from PC3 and HCT-15 cells. Genes.

[101] Özkoç M., Mutlu Altundag E. (2023). Antiproliferative effect of thymoquinone on human colon cancer cells: Is it dependent on glycolytic pathway?. Acibadem Univ. Saglik. Bilim. Derg..

[102] Thabet N.A., El-Khouly D., Sayed-Ahmed M.M., Omran M.M. (2021). Thymoquinone chemosensitizes human colorectal cancer cells to imatinib via uptake/efflux genes modulation. Clin. Exp. Pharmacol. Physiol..

[103] Ahmed D., Eide P.W., Eilertsen I.A., Danielsen S.A., Eknæs M., Hektoen M., Lind G.E., Lothe R.A. (2013). Epigenetic and genetic features of 24 colon cancer cell lines. Oncogenesis.

[104] Bertrand F.E., Angus C.W., Partis W.J., Sigounas G. (2012). Developmental pathways in colon cancer: Crosstalk between WNT, BMP, Hedgehog and Notch. Cell Cycle.

[105] Hon K.W., Zainal Abidin S.A., Othman I., Naidu R. (2021). The crosstalk between signaling pathways and cancer metabolism in colorectal cancer. Front. Pharmacol..

[106] Farooqi A.A., de la Roche M., Djamgoz M.B.A., Siddik Z.H. (2019). Overview of the oncogenic signaling pathways in colorectal cancer: Mechanistic insights. Semin. Cancer Biol..

[107] Farooqi A.A., Attar R., Xu B. (2022). Anticancer and anti-metastatic role of thymoquinone: Regulation of oncogenic signaling cascades by thymoquinone. Int. J. Mol. Sci..

[108] Zhang L., Bai Y., Yang Y. (2016). Thymoquinone chemosensitizes colon cancer cells through inhibition of NF-κB. Oncol. Lett..

[109] Kundu J., Choi B.Y., Jeong C.H., Kundu J.K., Chun K.S. (2014). Thymoquinone induces apoptosis in human colon cancer HCT116 cells through inactivation of STAT3 by blocking JAK2- and Src-mediated phosphorylation of EGF receptor tyrosine kinase. Oncol. Rep..

[110] Wirries A., Breyer S., Quint K., Schobert R., Ocker M. (2010). Thymoquinone hydrazone derivatives cause cell cycle arrest in p53-competent colorectal cancer cells. Exp. Ther. Med..

[111] Hsu H.H., Chen M.C., Day C.H., Lin Y.M., Li S.Y., Tu C.C., Padma V.V., Shih H.N., Kuo W.W., Huang C.Y. (2017). Thymoquinone suppresses migration of LoVo human colon cancer cells by reducing prostaglandin E2 induced COX-2 activation. World J. Gastroenterol..

[112] Rooney S., Ryan M.F. (2005). Effects of alpha-hederin and thymoquinone, constituents of *Nigella sativa*, on human cancer cell lines. Anticancer Res..

[113] Gali-Muhtasib H., Diab-Assaf M., Boltze C., Al-Hmaira J., Hartig R., Roessner A., Schneider-Stock R. (2004). Thymoquinone extracted from black seed triggers apoptotic cell death in human colorectal cancer cells via a p53-dependent mechanism. Int. J. Oncol..

[114] Gali-Muhtasib H., Kuester D., Mawrin C., Bajbouj K., Diestel A., Ocker M., Habold C., Foltzer-Jourdainne C., Schoenfeld P., Peters B. (2008). Thymoquinone triggers inactivation of the stress response pathway sensor CHEK1 and contributes to apoptosis in colorectal cancer cells. Cancer Res..

[115] Chen M.C., Lee N.H., Hsu H.H., Ho T.J., Tu C.C., Hsieh D.J., Lin Y.M., Chen L.M., Kuo W.W., Huang C.Y. (2015). Thymoquinone induces caspase-independent, autophagic cell death in CPT-11-resistant lovo colon cancer via mitochondrial dysfunction and activation of JNK and p38. J. Agric. Food Chem..

[116] Asfour W., Almadi S., Haffar L. (2013). Thymoquinone suppresses cellular proliferation, inhibits VEGF production and obstructs tumor progression and invasion in the rat model of DMH-induced colon carcinogenesis. Pharm. Pharmacol..

[117] Bardelčíková A., Šoltys J., Mojžiš J. (2023). Oxidative stress, inflammation and colorectal cancer: An overview. Antioxidants.

[118] Boakye D., Jansen L., Schöttker B., Jansen E.H.J.M., Schneider M., Halama N., Gào X., Chang-Claude J., Hoffmeister M., Brenner H. (2020). Blood markers of oxidative stress are strongly associated with poorer prognosis in colorectal cancer patients. Int. J. Cancer.

[119] Gackowski D., Banaszkiewicz Z., Rozalski R., Jawien A., Olinski R. (2002). Persistent oxidative stress in colorectal carcinoma patients. Int. J. Cancer.

[120] Vodicka P., Urbanova M., Makovicky P., Tomasova K., Kroupa M., Stetina R., Opattova A., Kostovcikova K., Siskova A., Schneiderova M. (2020). Oxidative damage in sporadic colorectal cancer: Molecular mapping of base excision repair glycosylases in colorectal cancer patients. Int. J. Mol. Sci..

[121] Al-Johar D., Shinwari N., Arif J., Al-Sanea N., Jabbar A.A., El-Sayed R., Mashhour A., Billedo G., El-Doush I., Al-Saleh I. (2008). Role of *Nigella sativa* and a number of its antioxidant constituents towards azoxymethane-induced genotoxic effects and colon cancer in rats. Phytother. Res..

[122] Jrah-Harzallah H., Ben-Hadj-Khalifa S., Almawi W.Y., Maaloul A., Houas Z., Mahjoub T. (2013). Effect of thymoquinone on 1,2-dimethyl-hydrazine-induced oxidative stress during initiation and promotion of colon carcinogenesis. Eur. J. Cancer.

[123] Harzallah H.J., Grayaa R., Kharoubi W., Maaloul A., Hammami M., Mahjoub T. (2012). Thymoquinone, the *Nigella sativa* bioactive compound, prevents circulatory oxidative stress caused by 1,2-dimethylhydrazine in erythrocyte during colon postinitiation carcinogenesis. Oxid. Med. Cell. Longev..

[124] Blondy S., David V., Verdier M., Mathonnet M., Perraud A., Christou N. (2020). 5-Fluorouracil resistance mechanisms in colorectal cancer: From classical pathways to promising processes. Cancer Sci..

[125] Pouya F.D., Gazouli M., Rasmi Y., Lampropoulou D.I., Nemati M. (2022). MicroRNAs and drug resistance in colorectal cancer with special focus on 5-fluorouracil. Mol. Biol. Rep..

[126] Gmeiner W.H., Okechukwu C.C. (2023). Review of 5-FU resistance mechanisms in colorectal cancer: Clinical significance of attenuated on-target effects. Cancer Drug Resist..

[127] Shibata J., Aiba K., Shibata H., Minowa S., Horikoshi N. (1998). Detection and quantitation of thymidylate synthase mRNA in human colon adenocarcinoma cell line resistant to 5-fluorouracil by competitive PCR. Anticancer Res..

[128] Lv L., Liu H.G., Dong S.Y., Yang F., Wang Q.X., Guo G.L., Pan Y.F., Zhang X.H. (2016). Upregulation of CD44v6 contributes to acquired chemoresistance via the modulation of autophagy in colon cancer SW480 cells. Tumour Biol..

[129] Chen J., Na R., Xiao C., Wang X., Wang Y., Yan D., Song G., Liu X., Chen J., Lu H. (2021). The loss of SHMT2 mediates 5-fluorouracil chemoresistance in colorectal cancer by upregulating autophagy. Oncogene.

[130] Romano G., Santi L., Bianco M.R., Giuffrè M.R., Pettinato M., Bugarin C., Garanzini C., Savarese L., Leoni S., Cerrito M.G. (2016). The TGF-β pathway is activated by 5-fluorouracil treatment in drug resistant colorectal carcinoma cells. Oncotarget.

[131] Longley D.B., Johnston P.G. (2005). Molecular mechanisms of drug resistance. J. Pathol..

[132] Schiavoni G., Gabriele L., Mattei F. (2013). The tumor microenvironment: A pitch for multiple players. Front. Oncol..

[133] Crea F., Nobili S., Paolicchi E., Perrone G., Napoli C., Landini I., Danesi R., Mini E. (2011). Epigenetics and chemoresistance in colorectal cancer: An opportunity for treatment tailoring and novel therapeutic strategies. Drug Resist. Updat..

[134] Fazzone W., Wilson P.M., LaBonte M.J., Lenz H.-J., Ladner R.D. (2009). Histone deacetylase inhibitors suppress thymidylate synthase gene expression and synergize with the fluoropyrimidines in colon cancer cells. Int. J. Cancer.

[135] Kensara O.A., El-Shemi A.G., Mohamed A.M., Refaat B., Idris S., Ahmad J. (2016). Thymoquinone subdues tumor growth and potentiates the chemopreventive effect of 5-fluorouracil on the early stages of colorectal carcinogenesis in rats. Drug Des. Dev. Ther..

[136] Eftekhar S.P., Kazemi S., Moghadamnia A.A. (2022). Effect of thymoquinone on pharmacokinetics of 5-fluorouracil in rats and its effect on human cell line in vitro. Hum. Exp. Toxicol..

[137] Ballout F., Monzer A., Fatfat M., Ouweini H.E., Jaffa M.A., Abdel-Samad R., Darwiche N., Abou-Kheir W., Gali-Muhtasib H. (2020). Thymoquinone induces apoptosis and DNA damage in 5-Fluorouracil-resistant colorectal cancer stem/progenitor cells. Oncotarget.

[138] Ndreshkjana B., Çapci A., Klein V., Chanvorachote P., Muenzner J.K., Huebner K., Steinmann S., Erlenbach-Wuensch K., Geppert C.I., Agaimy A. (2019). Combination of 5-fluorouracil and thymoquinone targets stem cell gene signature in colorectal cancer cells. Cell Death Dis..

[139] Norwood A.A., Tucci M., Benghuzzi H. (2007). A comparison of 5-fluorouracil and natural chemotherapeutic agents, EGCG and thymoquinone, delivered by sustained drug delivery on colon cancer cells. Biomed. Sci. Instrum..

[140] Zaki S.M., Waggas D.S. (2022). Protective effect of *Nigella sativa* and onion extract against 5-fluorouracil-induced hepatic toxicity. Nutr. Cancer.

[141] Madani F., Kazemi S., Shirafkan F., Lotfi M., Hosseini S.M., Moghadamnia A.A. (2023). Thymoquinone protects against 5-fluorouracil-induced mucositis by NF-κβ and HIF-1 mechanisms in mice. J. Biochem. Mol. Toxicol..

[142] Effenberger-Neidnicht K., Schobert R. (2011). Combinatorial effects of thymoquinone on the anti-cancer activity of doxorubicin. Cancer Chemother. Pharmacol..

[143] Khalife R., Hodroj M.H., Fakhoury R., Rizk S. (2016). Thymoquinone from *Nigella sativa* seeds promotes the antitumor activity of noncytotoxic doses of topotecan in human colorectal cancer cells in vitro. Planta Med..

